# A recurrent translocation is mediated by homologous recombination between HERV-H elements

**DOI:** 10.1186/1755-8166-5-6

**Published:** 2012-01-19

**Authors:** Karen E Hermetz, Urvashi Surti, Jannine D Cody, M Katharine Rudd

**Affiliations:** 1 Department of Human Genetics, Emory University School of Medicine, Atlanta, GA, USA; 2 Department of Pathology, University of Pittsburgh School of Medicine, Pittsburgh, PA, USA; 3 Department of Pediatrics, Chromosome 18 Registry and Research Society, University of Texas Health Science Center at San Antonio, San Antonio, TX, USA

**Keywords:** HERV-H, HERV, NAHR, translocation, t(4;18), recurrent translocation, 4q35.1, 18q22.3, 18q

## Abstract

**Background:**

Chromosome rearrangements are caused by many mutational mechanisms; of these, recurrent rearrangements can be particularly informative for teasing apart DNA sequence-specific factors. Some recurrent translocations are mediated by homologous recombination between large blocks of segmental duplications on different chromosomes. Here we describe a recurrent unbalanced translocation casued by recombination between shorter homologous regions on chromosomes 4 and 18 in two unrelated children with intellectual disability.

**Results:**

Array CGH resolved the breakpoints of the 6.97-Megabase (Mb) loss of 18q and the 7.30-Mb gain of 4q. Sequencing across the translocation breakpoints revealed that both translocations occurred between 92%-identical human endogenous retrovirus (HERV) elements in the same orientation on chromosomes 4 and 18. In addition, we find sequence variation in the chromosome 4 HERV that makes one allele more like the chromosome 18 HERV.

**Conclusions:**

Homologous recombination between HERVs on the same chromosome is known to cause chromosome deletions, but this is the first report of interchromosomal HERV-HERV recombination leading to a translocation. It is possible that normal sequence variation in substrates of non-allelic homologous recombination (NAHR) affects the alignment of recombining segments and influences the propensity to chromosome rearrangement.

## Background

Chromosome rearrangements play a major role in intellectual disability, birth defects, and autism [1-3], and many heterogeneous mechanisms have been implicated in the formation of chromosome rearrangements. Analyses of breakpoint junctions from a host of chromosome rearrangements have revealed signatures of homologous recombination (HR), nonhomologous end-joining (NHEJ), microhomology-mediated end joining (MMEJ), and break-induced replication (BIR) [4-6].

Recurrent chromosome abnormalities give us a unique opportunity to unravel specific factors involved in rearrangement, as their frequency and shared breakpoints indicate a rearrangement-prone genomic architecture. Deletions and duplications mediated by non-allelic homologous recombination (NAHR) are the most common class of recurrent constitutional chromosome rearrangements and are responsible for several genetic syndromes [7-9]. Such rearrangements are relatively easy to identify by paralogous genomic segments that are highly identical and typically hundreds of kilobases in size flanking breakpoint junctions [9-11]. Analysis of translocation breakpoints has shown that some recurrent translocations are also mediated by NAHR. For example, recurrent translocations between chromosomes 4p and 8p [12], 4q and 10q [13], and 4p and 11p [14,15] are known to be mediated by homologous recombination between large blocks of segmental duplications, whereas recurrent translocations between 11q and 22q [16] and 8q and 22q [17] are initiated by palindromic AT-rich sequences at breakpoints. However, most constitutional translocations are not recurrent, and their breakpoints lack significant sequence homology that would suggest NAHR [18-20].

NAHR between homologous interspersed repeats like LINE, *Alu*, and human endogenous retrovirus (HERV) elements can also lead to genomic rearrangements [21,22]. *Alu*-*Alu *recombination events have been described at multiple loci, giving rise to pathogenic deletions, duplications, and translocations [6,23-26]. Homologous recombination between HERV15 elements underlies the recurrent Y chromosome microdeletion that removes the azoospermia factor a (AZFa) region and causes male infertility [27-29]. Recently, a HERV-H-mediated deletion of chromosome 8q13.3 has been described in a child with heterozygous loss of the *EYA1 *gene and branchio-oto-renal syndrome [30]. Nevertheless, homologous recombination between HERV elements on *different *chromosomes has not been previously described as a mechanism for recurrent *translocations*.

HERVs make up ~3% of the human genome [31,32]; however, most copies have mutations and/or deletions that disrupt one or more of the ORFs, rendering the retrovirus inactive [33-35]. Though most HERVs are inactive as retrotransposons, they may spread through the genome via ectopic recombination processes. Phylogenetic studies of HERV-K elements have revealed signatures of intraelement gene conversion and recombination [36,37]. Thus, the density of HERVs and the sequence homogenization between copies make them ideal substrates for NAHR in the human genome.

Here we describe a recurrent translocation mediated by NAHR between HERVs on chromosomes 4q and 18q. Sequencing the breakpoint junctions in two unrelated individuals with similar translocations [38,39] revealed breakpoints within a few hundred basepairs (bp) of each other. Both sets of translocation breakpoints are located in HERV-H elements, and the orientation and sequence homology between recombining segments on 4q and 18q are consistent with a NAHR rearrangement mechanism.

## Results and discussion

In an earlier study, we identified two individuals (18q-82C and 18q-146C) with unbalanced translocations between the ends of the long arms of chromosomes 4 and 18. Both carry a derivative chromosome missing the end of chromosome 18q, with an additional copy of the end of 4q, as shown previously by array comparative genomic hybridization (CGH) [38,39]. Although two children are not enough for us to evaluate the phenotype associated with this unbalanced translocation comprehensively, some shared clinical features are worth noting. Patients 18q-82C and 18q-146C both exhibited developmental delays, auditory canal atresia, midface hypoplasia, microcephaly, and a broad nasal bridge. Parental studies revealed that 18q-82C carries a *de novo *translocation, derived from paternal chromosomes 4 and 18 [39]. Parents of 18q-146C were not available for study.

**Figure 1:**
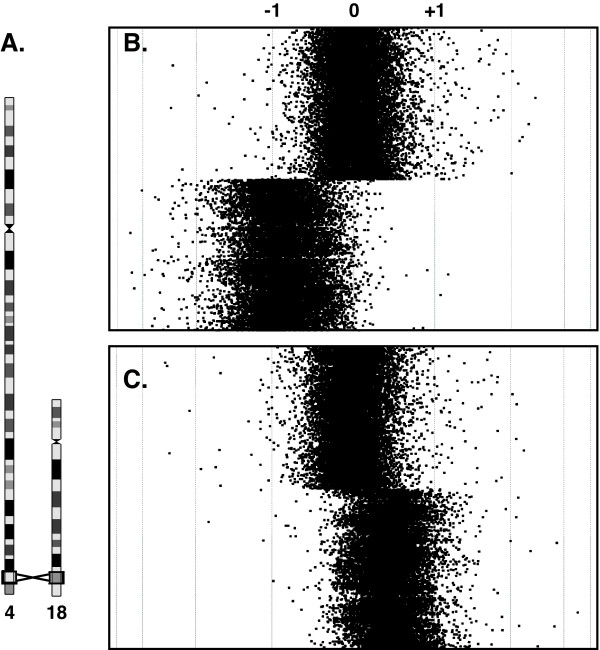
**High-resolution array CGH reveals the breakpoints of the unbalanced translocation from patient 18q-146C**. (**a**) The recurrent translocation occurs between 4q35.1 and 18q22.3, as indicated by the boxed chromosome bands. (**b**) The 6.97-Mb loss of 18q and (**c**) the 7.30-Mb gain of the end of 4q are shown. Averaged log_2 _ratios of probe signal intensities are shown (black dots). Dashed lines indicate log_2 _ratios of -1, 0, and +1. Similar array CGH results were obtained from patient 18q-82C's translocation.

Using high-resolution array CGH, we resolved the 4q and 18q breakpoints in both subjects. We designed a custom oligonucleotide array targeting the 5 Mb spanning the 4q and 18q breakpoints with a mean probe spacing of one oligonucleotide per 100 bp. Array CGH revealed the same breakpoints in both individuals: a 6.97-Mb loss of 18q and a 7.30-Mb gain of 4q (Figure 1).

**Figure 2:**
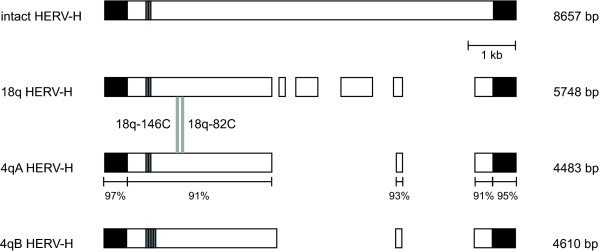
**HERV-H elements from chromosomes 4q and 18q, as compared to the intact HERV-H consensus sequence**. The HERV-H consensus is 8657 bp as represented in RepeatMasker [40]. HERVs and long terminal repeats (LTRs) are depicted as open and filled black rectangles, respectively. The 18q HERV-H sequence is derived from Build 36.1 (hg18) of the human genome assembly. HERV-H sequences from the 4qA and 4qB alleles were derived from 18q-82P. Sequence identity between the 4q and 18q HERV-Hs are shown across aligning segments. The 61-bp tandem repeats are represented as dark grey rectangles within the HERV-Hs. Sites of recombination between 4q and 18q that gave rise to translocations in 18q-82C and 18q-146C are shown as vertical grey lines.

Based on the array CGH data, we designed PCR primers to amplify across the breakpoint junctions of the derivative chromosomes 18 from patients 18q-82C and 18q-146C. We cloned and sequenced the breakpoint junctions to generate complete sequence across the two independent junction fragments (GenBank sequences 18q82C_junction and 18q146C_junction). In both translocations, the junction between chromosomes 18 and 4 lies in a HERV-H element (Figure 2). As represented in the reference genome (Build 36.1, hg18), the HERV-H elements on chromosomes 4q and 18q are 4.6 kilobases (kb) and 5.7 kb, respectively, and are ~92% identical overall.

**Figure 3:**
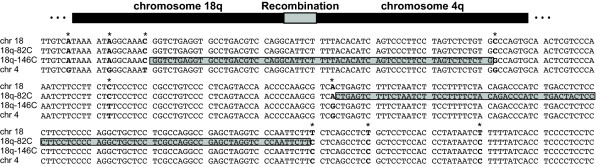
**Sequence alignment of the translocation junction sequences from patients 18q-82C and 18q-146C**. Sequences for chromosomes 18 (chr 18) and 4 (chr 4) were derived from Build 36.1 (hg18) of the human genome assembly. SNPs that are specific to chromosome 4 or chromosome 18 are shown in bold and marked by asterisks. The sites of recombination between chromosomes 18q and 4q are highlighted in grey.

We aligned our junction sequences from patients 18q-82C and 18q-146C to sequence from the 4q and 18q HERV-H elements in the human genome assembly (Build 36.1, hg18). Recombination for both translocations occurred in an ~three-kb region that is 91% identical between the 4q and 18q HERV-Hs (Figure 2). Though the HERV-Hs are highly identical, chromosome-specific SNPs distinguish the 18q and 4q sides of the translocation junctions and allow us to further resolve the sites of recombination. The sites of recombination mediating the translocations in patients 18q-82C and 18q-146C are ~150 bp apart (Figure 3).

It is possible that some copies of the 4q and 18q HERV-Hs share more homology than represented in the human genome assembly, which may affect the propensity of the two chromosomes to recombine. There are reports of sequence variation in HERVs at several loci, which may occur via gene conversion or transposition processes [29,36,37]. To capture the actual HERV-H sequences that recombined to form the translocations in patients 18q-82C and 18q-146C, it was necessary to sequence the parental 4q and 18q alleles. Microsatellite analysis of 18q-82C and his parents revealed that the *de novo *translocation was paternal in origin [39]. We sequenced both alleles of the HERV-H elements on 4q and 18q in 18q-82C's father (18q-82P). The two 18q HERV-H alleles in 18q-82P were 99.5% identical to the 18q HERV-H in the reference genome, with no significant differences between the two alleles. However, 18q-82P has two different 4q HERV-H alleles, described here as 4qA (GenBank sequence 82Pchr4HERVHA) and 4qB (GenBank sequence 82Pchr4HERVHB).

The HERV-H elements on 4qA and 4qB are 99.0% identical, but 4qB has a 122-bp duplication not present in 4qA (Figure 2). The duplication is made up of a 61-bp tandem repeat present in two copies and four copies on the 4qA and 4qB alleles, respectively. The human reference genome (Build 36.1, hg18) corresponds to the 4qB allele, including the 122-bp duplication. The 122-bp duplication is not present in the 18q HERV-H or in the HERV-H consensus sequence [40], consistent with the duplication being a new event that arose on the 4qB allele.

To determine the frequency of the 4qA and 4qB alleles in the human population, we designed a genotyping assay that distinguishes the two alleles. We performed a nested PCR that specifically amplifies a six-kb region, including the entire 4q HERV-H in the first PCR, followed by a second PCR that amplifies the region around the 122-bp duplication. This results in a 326-bp band for the 4qA allele and a 446-bp band for the 4qB allele. We genotyped DNA from 99 Caucasians obtained from the Coriell Cell Repository (Human Variation Panel HD100CAU), representing 198 4q alleles, of which 37 (18.7%) were 4qA and 161 (81.3%) were 4qB (Additional file 1). We also genotyped 62 samples from 10 populations included in the Human Genome Diversity Panel (HGDP). Allele frequencies were not significantly different between geographic populations, and as a group, there were 26 (21.0%) 4qA alleles and 98 (79.0%) 4qB alleles (Additional file 1). Thus, the 4qA allele carried by 18q-82P is the minor allele, and the 4qB allele in the reference genome is the major allele.

## Conclusions

Recurrent copy number variations (e.g., deletions, duplications, and translocations) provide mechanistic insight into the etiology of chromosome rearrangements. We sequenced the breakpoint junctions of two independent translocations with nearly identical breakpoints on chromosomes 4q and 18q. Analysis of the recombining segments revealed that the breakpoints lie in HERV-H elements that are 92% identical. NAHR between other HERVs has been found to underlie interstitial deletions of the Y chromosome and chromosome 8; in both cases, the recombining HERVs were ~94% identical [27-30].

NAHR between larger segmental duplications causes the most common microdeletion and microduplication syndromes. In these cases, recombining segmental duplications range from 10 kb to hundreds of kilobases in size and are typically 95% identical or greater [10,41-43]. NAHR between smaller substrates with greater sequence divergence, such as the HERV-Hs described here, is probably less frequent than NAHR between large segmental duplications. However, sequence variation in recombining segments could also impact rearrangement frequency. Polymorphism in the HERV15 elements that mediate the recurrent AZFa microdeletion leads to better sequence alignment between the recombining regions on the Y chromosome and is predicted to predispose to microdeletion [29]. It is possible that sequence variation in the 4q HERV-H also affects its propensity to recombine with the 18q HERV-H. Our study of two recurrent translocations is not comprehensive enough to draw conclusions about HERV-H sequence variation and recombination frequency. However, the lack of the 122-bp duplication in the 4qA HERV-H makes it more similar to the 18q HERV-H, which may be important for recombination between the two chromosomes. Furthermore, the fact that 18q-82P carries a 4qA allele is consistent with recombination between the 4qA HERV-H and the 18q HERV-H to give rise to patient 18q-82C's translocation. Translocations in patients 18q-82C and 18q-146C both occurred in the same region of the HERV-H that is polymorphic between the 4q alleles (Figure 2), and the 122-bp duplication alters the sequence alignment between this part of chromosomes 4q and 18q.

It is important to point out that we only recognized the signatures of HERV-HERV NAHR at the translocation breakpoints of patients 18q-82C and 18q-146C after sequencing the breakpoint junctions. Inferring chromosomal rearrangement mechanisms from lower-resolution approaches (e.g., array CGH only) is likely to underestimate the frequency of NAHR-mediated events between shorter homologous segments and only detect NAHR between large blocks of segmental duplication. Furthermore, NAHR between homologous interspersed repeats, such as *Alu*s, LINEs, and HERVs, would be overlooked by strategies focusing on recombination between segmental duplications [15]. We have previously detected a translocation that is the product of interchromosomal LINE-LINE recombination between L1PA2s on chromosomes 6 and 16 that are 96% identical over ~six kb by sequencing across the translocation breakpoint junction [6]. In addition, sequencing of normal copy number variation breakpoints has uncovered signatures of intrachromosomal NAHR [44,45]. Future sequencing-based studies of other chromosome rearrangements will likely capture more NAHR events between shorter homologous segments, which would give us a better understanding of the requirements for interchromosomal and intrachromosomal NAHR in the human genome.

## Methods

### Participants

We obtained informed consent from individuals with chromosome 18 abnormalities and their families. The human subjects protocol was approved by the Institutional Review Board of the University of Texas Health Science Center at San Antonio.

### Array CGH

Using a 244K platform from Agilent Technologies (Santa Clara, CA), we designed a custom two-plex array covering the five-Mb regions spanning the previously described breakpoints on chromosomes 4q and 18q [39], with a mean probe spacing of one oligonucleotide per 100 basepairs (bp). Oligonucleotides were designed using Agilent's eArray program (https://earray.chem.agilent.com/earray/). To minimize non-unique oligonucleotides that would not be informative in array CGH, we performed a high definition (HD) probe search to prefer existing "catalog probes" and used the most stringent "similarity score filter" designed to select probes that hybridize to only one genomic location. The unique identifier (AMADID) for the array design is 021748; this design is available upon request.

Lymphoblastoid cell lines derived from 18q-82C, 18q-82P, and 18q-146C were established previously [39]. We extracted genomic DNA from cell lines using the Gentra Puregene DNA Extraction Kit (Qiagen, Valencia, CA). Subject DNA was co-hybridized with reference DNA from the GM15510 cell line (Coriell Cell Repositories, Camden, NJ). Arrays were scanned using a GenePix 4000B scanner (Molecular Devices, Sunnyvale, CA), and signal intensities were evaluated using Feature Extraction Version 9.5.1.1 software (Agilent Technologies, Santa Clara, CA). We used DNA Analytics Version 4.0 software (Agilent Technologies, Santa Clara, CA) to analyze the array data and call breakpoints.

### Breakpoint PCR

Starting with breakpoints identified by array CGH, we designed PCR primers to amplify across the translocation breakpoint junctions in 18q-82C and 18q-146C. The chromosome 18-specific primer is 5'-TCAACTGTAGAAGAGGTCAGAGCTCCCCTA-3', and the chromosome 4-specific primer is 5'-GGTCAATGATCCGGAGGGTTCTGGATG-3'. We performed PCR using TaKaRa Ex *Taq *polymerase (Clontech Laboratories, Inc., Madison, WI) with 1× PCR buffer, 0.2 mM dNTP, 8 pmol of each primer, and 50-100 ng of DNA template. PCR conditions were as follows. 94°C for 1 min; 10 cycles at 94°C for 30 s, 66°C for 1 min, decreasing 0.5°C per cycle, and 72°C for 7 min; 20 cycles at 94°C for 30 s, 61°C for 1 min, and 72°C for 7 min; and a final extension at 72°C for 10 min. Bands were visualized via gel electrophoresis on a 1% agarose gel.

### Nested PCR

We designed a nested PCR to specifically amplify the HERV-Hs on 4qA and 4qB. The first PCR amplifies a 6-kb region larger than the 4q HERV-Hs using the following primers: 5'-GATCATTTTGTCAATGAAATCTCACAAGAGGGC-3' and 5'-GGTCAATGATCCGGAGGGTTCTGGATG-3'. The PCR reagents were the same as described above, except for the addition of betaine (0.7 M final concentration in 50 μl PCR). Conditions for the first PCR were: 94°C for 1 min; 10 cycles at 94°C for 30 s, 65°C for 1 min, decreasing 0.5°C per cycle, and 72°C for 7.5 min; 30 cycles at 94°C for 30 s, 60°C for 1 min, and 72°C for 7.5 min; and a final extension at 72°C for 10 min.

We diluted amplicons from the first PCR 1:1000 to use as template in the second PCR. The second PCR amplifies a 326-bp or a 446-bp product from the 4qA and 4qB alleles, respectively, using the following primers: 5'-CACCTGCTTTGGTCCTTCAC-3' and 5'-ACTTTCCCCTCTCCCAGAAA-3'. Conditions for the second PCR were: 94°C for 1 min; 35 cycles at 94°C for 20 s, 55°C for 10 s, and 72°C for 10 s; and a final extension at 72°C for 1 min. Bands were visualized via gel electrophoresis on a 1% agarose gel.

### Sequence analysis

We purified PCR products from agarose gels using the QIAquick gel extraction kit (Qiagen, Valencia, CA), and cloned them into a TOPO-TA vector following the manufacturer's protocol (Invitrogen, Carlsbad, CA). We transformed the ligated construct into SURE 2 Supercompetent Cells (Agilent Technologies, Cedar Creek, TX) following the manufacturer's protocol. We propagated plasmids in recombination-deficient SURE 2 *Escherichia coli *to prevent rearrangement of the cloned insert.

We purified plasmid DNA (Qiagen Miniprep kit, Valencia, CA) and submitted plasmids for Sanger sequencing (Beckman Coulter Genomics, Danvers, MA). DNA sequences were analyzed by comparing reads to the human genome reference assembly (NCBI36/hg18) using the BLAT tool [46] on the UCSC Genome Browser (http://genome.ucsc.edu/). Junction sequences from 18q-82C (18q82C_junction) and 18q-146C (18q146C_junction) have been deposited in GenBank. The breakpoint junctions on chromosome 4 correspond to positions chr4:183974152-183974212 (18q-146C) and chr4:183974285-183974380 (18q-82C) and the breakpoint junctions on chromosome 18 correspond to positions chr18:69144350-69144410 (18q-146C) and chr18:69144483-69144578 (18q-82C) of the NCBI36/hg18 build of the human genome.

We also cloned and sequenced the products of the first round of nested PCR from 82P to characterize the entire 6-kb HERV-H regions from the 4qA and 4qB alleles. These sequences have been deposited in GenBank as 82Pchr4HERVHA and 82Pchr4HERVHB. We aligned the intact HERV-H sequences from 4qA and 4qB to the HERV-H consensus as represented in RepeatMasker [40] and analyzed the 61-bp tandem repeat within the HERV-H using Tandem Repeats Finder (TRF), http://tandem.bu.edu/trf/trf.html[47].

## Competing interests

The authors declare that they have no competing interests.

## Authors' contributions

KEH performed the array CGH, breakpoint sequencing, and genotyping experiments. KEH and MKR performed the sequence alignments and analyzed the data. MKR drafted the manuscritpt. US and JDC participated in the design of the study and the manuscript. All authors have read and approved the manuscript.

## Supplementary Material

Additional file 1 **Gentoype results of the 4qA and 4qB alleles in the population**. Click here for file
